# Hippo Signaling in the Liver – A Long and Ever-Expanding Story

**DOI:** 10.3389/fcell.2019.00033

**Published:** 2019-03-12

**Authors:** Saumya Manmadhan, Ursula Ehmer

**Affiliations:** Department of Medicine II, Klinikum Rechts der Isar, Technische Universität München, Munich, Germany

**Keywords:** hippo, yap, liver, hepatocellular carcinoma, HCC, cholangiocarcinoma, fibrosis, steatohepatitis

## Abstract

The first description of Hippo signaling in mammals a little more than 10 years ago showed a striking phenotype in the liver, linking the role of this signaling pathway to organ size control and carcinogenesis. Even though Hippo signaling has been extensively studied in the liver and other organs over the recent years, many open questions remain in our understanding of its role in hepatic physiology and disease. The functions of Hippo signaling extend well beyond cancer and organ size determination: components of upstream Hippo signaling and the downstream effectors YAP and TAZ are involved in a multitude of cell and non-cell autonomous functions including cell proliferation, survival, development, differentiation, metabolism, and cross-talk with the immune system. Moreover, regulation and biological functions of Hippo signaling are often organ or even cell type specific – making its role even more complex. Here, we give a concise overview of the role of Hippo signaling in the liver with a focus on cell-type specific functions. We outline open questions and future research directions that will help to improve our understanding of this important pathway in liver disease.

## Mammalian Hippo Signaling – It All Started in the Liver

Several years after the identification and characterization of individual Hippo pathway members in *Drosophila* and mammals, the importance of Hippo signaling in the liver became evident with a striking phenotype: overexpression of YAP or expression of activated YAP resulted in dramatic overgrowth of the liver, identifying Hippo signaling as an important determinant in organ size control (Camargo et al., 2007; Dong et al., 2007). Rapid development of hepatocellular carcinoma (HCC) upon YAP overexpression further confirmed a potent oncogenic role of this protein (Dong et al., 2007). More recently, the investigation of Hippo signaling in non-parenchymal liver cells, including hepatic stellate cells (HSC) and liver sinusoidal endothelial cells (LSEC) has brought insight into the complex interplay between different hepatic cell types with profound impact on the pathophysiology of liver disease. Here, we provide an overview of Hippo signaling in the liver including recent advances and open questions along with future directions in the field.

### Hippo Regulators Restrict Proliferation and Maintain Differentiation in Hepatocytes

**FIGURE 1:**
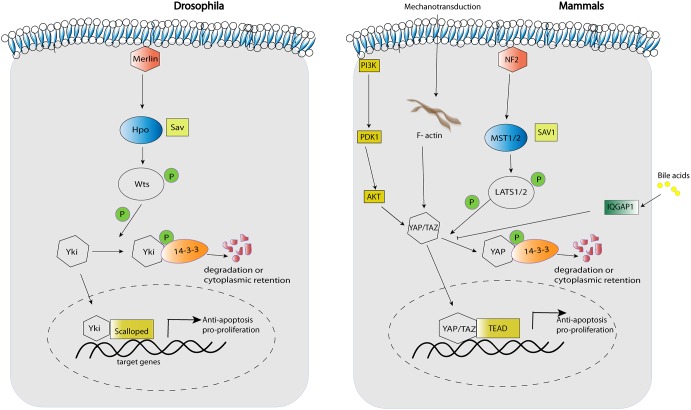
Key components of the Hippo pathway in Drosophila and mammals.

Soon after the discovery of YAP function in murine liver, MST1 and MST2 protein kinases were confirmed as upstream Hippo pathway regulators that restrict YAP activation, tissue overgrowth, and carcinogenesis (Figure 1; Zhou et al., 2009; Lu et al., 2010; Song et al., 2010). In the same line, hepatic inactivation of the MST1/2-adaptor protein SAV1/WW45 resulted in YAP-associated cell proliferation and mutant mice ultimately developed tumors with characteristics of HCC and intrahepatic cholangiocarcinomas (ICC) (Lee et al., 2010; Lu et al., 2010). The conditional knock-out of *Nf2*, the mammalian homolog of the upstream Hippo regulator *Merlin*, led to a reduction in Lats1/2 phosphorylation and thereby activation of YAP, resulting in hepatic overgrowth and liver tumor development (Benhamouche et al., 2010; Zhang et al., 2010). Importantly, these findings established NF2 as a negative regulator of YAP and confirmed that a large part of the *Drosophila* Hippo pathway is conserved in mammals (Figure 1).

In all of these models, conditional inactivation of Hippo pathway genes was achieved by using either *CAGGCre-ER* transgenic mice (Zhou et al., 2009; Song et al., 2010) or an *Albumin*-driven *Cre* (Benhamouche et al., 2010; Lu et al., 2010; Zhang et al., 2010), which is also active in fetal hepatoblasts that give rise to bile duct cells. Mutant mice showed varying degrees of hepatocyte proliferation but also exhibited proliferation and expansion of a hepatic cell population with small nuclei around the portal triad, so-called “oval cells.” These cells were long considered to function as bipotent liver progenitor cells that can differentiate into hepatocytes and bile duct cells under certain conditions such as severe hepatocyte damage – a hypothesis that has been challenged by recent research (Tanimizu and Mitaka, 2014). The expansion of oval cells and the development of both HCC and ICC initially led to the speculation that tumors in Hippo pathway-inactivated models arise from these potential bipotent progenitor cells. However, recent studies suggest that these phenotypes arise from trans-differentiation of mutant hepatocytes and deregulated biliary morphogenesis (Yimlamai et al., 2014; Benhamouche-Trouillet et al., 2018). Several hepatocyte-specific transfection models can trigger the development of tumors with mixed differentiation: overexpression of YAP as well as inactivation of the upstream Hippo regulator Nf2 mediated by AAV-Cre induces de-differentiation of hepatocytes toward a progenitor-like phenotype (Yimlamai et al., 2014). Additionally, hydrodynamic tail vein injection of transposon-based expression constructs for constitutively active YAP and PIK3CA – the catalytic subunit of PI3K – resulted in formation of liver tumors with hepatocellular, cholangiocellular, or mixed HCC/ICC differentiation. In this model, tumors were characterized by activation of mTORC1/2, ERK/MAPK, and Notch pathways (Li et al., 2015). To date, the molecular basis for the cooperation between PI3K and YAP signaling in liver cancer is not well understood, but could be mediated be PI3K-induced upregulation of CD166, a cell surface protein that has been shown to positively regulate YAP activity (Ma et al., 2014). On the other hand, data from breast epithelial cells and colon cancer cells indicate that PI3K/PDK1/AKT signaling promotes YAP activity via LATS-dependent and -independent mechanisms (Zhao et al., 2018). However, if this mechanism is conserved in liver cancer and how it relates to cellular differentiation remains to be investigated. From what is known to date, YAP – and possibly other oncogenic pathways such as PI3K signaling – not only seem to promote proliferation and tumorigenesis in general, but also oncogenic plasticity of hepatocytes with trans-differentiation toward a progenitor-like or even biliary phenotype (Yimlamai et al., 2014; Fitamant et al., 2015; Font-Burgada et al., 2015; Patel et al., 2017). Notch signaling – a critical pathway in bile duct development – is a key candidate for triggering this differentiation switch. Of note, Notch signaling can be activated though upregulation of the YAP target genes such as *Notch2* and *Jag1* (Jeliazkova et al., 2013; Tschaharganeh et al., 2013; Yimlamai et al., 2014; Wu et al., 2017; Zhang S. et al., 2018). However, a study using liver-specific knock-out of Hippo pathway kinases LATS1 and LATS2 did not confirm a role of Notch signaling but indicated that active TGFβ signaling downstream of YAP induces trans-differentiation into bile duct cells (Lee et al., 2016). Additionally, YAP promotes the binding of transcriptional regulators HNF4A and FOXA2 to embryonic enhancer sites during hepatocyte differentiation to increase transcription of embryo-specific genes – a mechanism that could be hijacked in transformed cells to promote dedifferentiation and oncogenesis (Alder et al., 2014). Taken together, a complex pattern of oncogenic pathway interactions determines hepatocyte plasticity and tumor differentiation upon Hippo pathway inactivation that we are only beginning to understand.

## Hippo Signaling in Bile Duct Cells – Same but Different

In addition to promoting biliary differentiation in hepatocytes, YAP signaling is of considerable relevance bile ducts cells. High cytoplasmic and nuclear YAP expression in cholangiocytes in human and murine livers suggest that YAP activity is of relevance in bile duct cells, but functional studies dissecting the role of Hippo signaling in cholangiocytes are limited. (Bai et al., 2012; Patel et al., 2017), Nevertheless, several interesting observations in *Albumin-Cre*-based conditional knock-out models that activate Cre in precursors of both hepatocytes and cholangiocytes were made (Geisler et al., 2008): conditional knock-out of YAP alone or of both YAP and its ortholog TAZ – also known as WWTR1 – results in defects in bile duct morphogenesis with irregularly shaped and deformed intrahepatic bile ducts (Zhang et al., 2010; Lu et al., 2018), while knock-out of *Nf2* resulted in the expansion of biliary structures (Benhamouche-Trouillet et al., 2018).

In humans, YAP is activated in ductular reactions in cholestatic liver disease and in non-alcoholic steatohepatitis (Bai et al., 2012; Anakk et al., 2013; Machado et al., 2015). In cholestatic liver damage after bile duct ligation in mice, this reactive proliferation and expansion of bile duct cells is markedly reduced in YAP-deficient livers – indicating that YAP activity is required for the formation of ductular reactions. In adult mice, genetic inactivation of YAP using the inducible *Mx-Cre* system results in profound hepatocyte damage and reduced compensatory proliferation of hepatocytes and bile duct cells, which is associated with increased mortality in comparison to wild type mice (Bai et al., 2012). Mechanistically, accumulation of bile acids in cholestatic liver disease seems to be an important activator of YAP downstream of the scaffolding protein IQGAP1, which is induced by bile acids – a mechanism that might also play a role in carcinogenesis (Anakk et al., 2013). Mechanistically, there is considerable evidence that IQGAP1 can directly bind to YAP to alter its transcriptional activity (Sayedyahossein et al., 2016). However, this interaction seems to be mostly inhibitory, indicating that a different, so far unknown mechanism might be responsible for IQGAP1-mediated YAP activation in the liver. In human cholangiocarcinoma, activated YAP is associated with poor prognosis, chemoresistance, angiogenesis, and chromosomal instability (Marti et al., 2015; Wu et al., 2016; Rizvi et al., 2018), and high TAZ expression correlates with a decreased survival after tumor resection (Xiao et al., 2016). In mice, expression of activated YAP and AKT in hepatocytes is sufficient to induce cholangiocarcinoma dependent on AKT/mTOR signaling, which likely cooperates with YAP in tumorigenesis and possibly promotes YAP activity (Zhang et al., 2017; Zhao et al., 2018). Furthermore, YAP is important for the maintenance of a cholangiocyte phenotype, as inhibition of YAP by overexpression of either LATS2 or of a dominant-negative variant of TEAD2 resulted in decreased expression of cholangiocyte markers (Zhang S. et al., 2018).

## Liver Regeneration – on the Good Side of YAP/TAZ

It has long been a mystery how the restoration of liver size almost exactly to its original volume is reliably achieved after liver resection. Hippo signaling seemed to be the ideal candidate for a pathway that governs controlled activation – and cessation – of hepatocyte proliferation during regeneration. Indeed, there is a robust activation of downstream YAP/TAZ about 24 h after partial hepatectomy followed by an activation of inhibitory Hippo kinases possibly to restrict excessive YAP/TAZ activation and halt proliferation once the liver approaches its original volume (Grijalva et al., 2014; Loforese et al., 2017; Lu et al., 2018). Liver-specific knock-out of YAP and TAZ results in impaired liver regeneration and delays restoration of liver mass (Lu et al., 2018), confirming the importance of Hippo signaling in liver regeneration. Contrarily, YAP and TAZ are not mandatory for completion of the regeneration process as alternative pathways can substitute for their activity to ensure liver regeneration – albeit with substantial delay (Michalopoulos, 2017). In fetal development, the *Alb-Cre* mediated conditional knockout of YAP in hepatocytes and cholangiocytes starting from embryonic day 13.5 resulted mainly in defective intrahepatic bile duct development – but interestingly in no overt phenotype in hepatocytes up to 8 weeks after birth (Zhang et al., 2010). In part, this finding has been attributed to rescue of YAP function by increased activity of its ortholog TAZ, similar to phenotypes overserved in zebrafish (Yi et al., 2018). But strikingly, deletion of both YAP and TAZ by *Albumin-Cre* leads to development of an almost-normal liver – and paradoxically results in an increase in liver size. This unexpected phenotype can be explained by an increase in hepatocyte damage with subsequent activation of other pro-proliferation pathways that are unable to control hepatic mass as tightly as Hippo signaling (Zhang et al., 2010; Lu et al., 2018). These findings highlight that pathway activation and interaction in embryonic development and during liver regeneration is characterized by a high plasticity to adopt to functional deficiency of major pro-proliferative pathways such as YAP/TAZ signaling. However, this functional redundancy does not seem to work as efficiently once the mice progress beyond a certain age: Older or diseased livers often fail to sufficiently regenerate as they are not able maintain adequate proliferative signaling. To some extent, this regeneration defect is due to hyperactive Hippo signaling – which is not sufficiently rescued by compensatory activation of other pro-proliferative pathways for reasons that are no completely understood to date. Silencing MST kinases by liposome-encapsulated siRNA restored expression of YAP target genes and improved liver regeneration in older animals (Loforese et al., 2017). In another study, a novel MST2 inhibitor reduced apoptosis and liver damage in acute aminacetophen-induced liver failure. Strikingly, a similar effect including subsiding fibrosis was observed in chronic liver injury after bile duct ligation or repeated CCl_4_-administration, respectively (Fan et al., 2016). Targeting upstream hippo kinases in human acute or chronic liver failure to restore regeneration is a promising approach with high clinical relevance – but must be weighed against the risk for oncogenic transformation especially in cirrhotic livers that inherently have a high risk for HCC development.

## Liver Fibrosis – the Scarring Face of YAP and TAZ

Hepatic stellate cells are the major cell type involved in liver fibrosis, but also play a critical role in acute repair after liver injury or partial hepatectomy (Kordes et al., 2014). Chronic liver damage triggers persistent activation of stellate cells with secretion and accumulation of excessive extracellular matrix (ECM) proteins, trans-differentiation of quiescent HSCs into myofibroblasts, cumulating in the formation of fibrotic scar tissue. The outcome of this dysregulated repair process is dictated by the molecular drivers that control HSC activation, which are therefore considered as candidates for targeted therapies. Recently, Hippo signaling has been identified as an important pathway in stellate cell activation. YAP is activated in HSCs during acute liver regeneration after partial hepatectomy or ischemia-reperfusion injury, and correspondingly in chronic injury in human fibrotic livers as well as in CCl_4_-induced fibrosis in mice (Mannaerts et al., 2015; Swiderska-Syn et al., 2016; Konishi et al., 2018). Importantly, sustained YAP activation in liver fibrosis is associated with an increase in ECM proteins and tissue stiffness (Dechene et al., 2010). The influence of matrix stiffness on YAP signaling is well established and mechanical cues are increasingly recognized as a mediator of pathological YAP activation *in vivo*: Increased matrix stiffness is associated with activation of cell surface receptors, that regulate F-actin polymerization in a RhoA-dependent manner to promote YAP/TAZ nuclear translocation and activation – probably providing an the molecular basis for YAP activation in HSCs and hepatocytes in fibrosis and possibly increasing carcinogenesis in cirrhotic livers (Dupont et al., 2011; Zhubanchaliyev et al., 2016). In acute liver injury, other pathways such as Hedgehog signaling likely contribute to YAP activation. After partial hepatectomy, mice with HSCs deficient for functional Hedgehog signaling show impaired activation of YAP in stellate cells and also in hepatocytes (Swiderska-Syn et al., 2016) – indicating that Hedgehog/YAP signaling mediates an important cross-talk between these cell types to ensure sufficient regeneration. While HSC activity is important for hepatic integrity following acute injury, sustained activation is deleterious in chronic liver damage – making YAP a potential target to prevent HSC activation and progression of fibrosis. Importantly, pharmacological inhibition of YAP/TEAD by verteporfin remarkably reduced HSC activation and impeded fibrogenesis that normally occurs after CCl_4_-treatment in mice (Mannaerts et al., 2015; Martin et al., 2016).

Other non-parenchymal cells with an essential role in liver injury are liver sinusoidal endothelial cells (LSECs), that have been shown to influence regeneration and fibrosis through angiocrine signaling to stellate cells in acute and chronic liver damage (Ding et al., 2014; Poisson et al., 2017). Co-culture experiments revealed that differentiated LSECs maintain HSC quiescence and that a dysregulated crosstalk between hepatocytes, LSECs, and HSCs in chronic liver injury contributes to fibrosis (Poisson et al., 2017). Interestingly, LSECs are also important in maintaining cell integrity after YAP activation in single hepatocytes. Combined hepatocyte and LSEC damage induced by ethanol – but not damage of hepatocytes or LSECs alone – changes the fate of YAP-activated hepatocytes from proliferation to apoptosis (Miyamura et al., 2017). YAP activation in LSECs has been shown to govern protein expression of HIF-1α and VEGF-A to promote angiogenesis in fibrosis – a process blocking nutrient transport from sinusoids and exacerbating the liver injury (Elpek, 2015; Zhang C. et al., 2018). Aside from triggering neo-angiogenesis in the diseased liver, it is highly likely that YAP/TAZ also play a major role in hepatic blood vessel formation during liver development, including in endothelial cell (EC) sprouting and junction maturation (Park and Kwon, 2018). However, a relevant expression of YAP/TAZ could not be detected in hepatic ECs at P5 and P12, which is in contrast to endothelial cells in developing retina and brain at the same time points – tissues where YAP and TAZ are functionally required for proper angiogenesis (Kim et al., 2017a). While this could indicate that YAP/TAZ signaling might not be relevant in liver angiogenesis, it is more likely that activation of YAP and TAZ does occur at earlier time points during liver development, where EC formation starts as early as E9.5 (Zhao and Duncan, 2005). If activation of YAP/TAZ signaling in endothelial cells plays a significant role in fibrosis and other liver diseases, however, remains to be investigated.

With the emerging importance of Hippo signaling in HSC, LSECs, and possibly endothelial cells in fibrosis and injury, any considerations to therapeutically target this pathway should take into account that liver regeneration will likely be impaired – possibly exacerbating liver injury in the long run.

## Steatohepatitis – a Link to Metabolism and Inflammation

In any tissue, cell proliferation and growth are dependent on nutrient availability. In this context, regulation of pro-proliferative YAP and TAZ by cellular metabolism is likely. Indeed, low energy levels trigger inhibition of YAP and TAZ, while high levels of glucose and fatty acids activate downstream YAP/TAZ transcription. On the other hand, YAP/TAZ themselves have been shown to promote key metabolic processes such as glycolysis to ensure an adequate energy supply in proliferating cells (Koo and Guan, 2018). In the liver, excess availability of nutrients can lead to steatosis and steatohepatitis (non-alcoholic fatty liver disease or NAFLD), in which fat accumulation results in inflammation and destruction hepatocytes, promoting the development of cirrhosis and liver cancer.

In murine models of NAFLD and in samples of human steatohepatitis an increase of YAP/TAZ levels can be observed – mainly in regenerative ductular reactions. In mice, viral expression of activated TAZ promotes inflammation in the liver, NAFLD, and tumor formation, linking TAZ – but not YAP – to an inflammatory signature in tumor development (Wang et al., 2016; Hagenbeek et al., 2018). Mechanistically, TAZ/TEAD directly activate Hedgehog signaling with increased expression of pro-fibrinogenic factors in HSC, including osteopontin, *Timp1*, and *Col1a1* (Machado et al., 2015; Wang et al., 2016). Interestingly, a transposon screen in mice identified mutations in the Hippo adaptor protein *Sav1* in NAFLD-associated tumors, but not those that developed in viral hepatitis (Kodama et al., 2018). One mechanism how steatosis could promote YAP/TAZ activation is through the obesity-associated protein JCAD that inhibits upstream LATS2 (Ye et al., 2017). To date, the differential role of YAP and TAZ is far from understood and most models of liver disease focus on the role of YAP. However, the abovementioned studies show that TAZ might have a unique role in steatohepatitis and the development of NAFLD-associated fibrosis.

## Liver Cancer – the Oncogenic Roots of YAP

Cirrhosis and chronic inflammation in the liver are leading risk factors for liver cancer (Massarweh and El-Serag, 2017). While YAP activation is an early event in HCC development (Perra et al., 2014), mutations within the Hippo pathway are rare – indicating for alternative regulatory pathways to promote YAP signaling. The search for upstream Hippo signaling regulators led to the identification of a variety of regulatory mechanisms ranging from mechanotransduction over GPCR signaling to interacting pathways, most prominently the Wnt signaling pathway (Meng et al., 2016; Dobrokhotov et al., 2018; Liu et al., 2018).

Given the increase in liver stiffness that occurs in cirrhosis, mechanical inputs are key candidates for oncogenic YAP activation that contributes to fibrosis progression and liver cancer. Mechanistically, several membrane-associated proteins such as Nf2/Merlin, scaffolding proteins of the angiomotin family, and WWC proteins have been shown to positively and – for certain members of the angiomotin family – negatively regulate Hippo signaling (Zhang et al., 2010; Li et al., 2012; Yi et al., 2013; Moleirinho et al., 2017; Hermann et al., 2018) – however, the relevance of these proteins in cirrhosis is unknown to date. Several interacting pathways, including Wnt and Notch signaling, play a role in the control of oncogenic YAP in liver cancer. In hepatocellular carcinoma, Notch signaling cannot only be activated by YAP, but also maintains a positive feedback loop in Mst1/2-deficient livers to enhance YAP signaling and tumorigenesis. In contrast, Wnt signaling has been shown to inhibit hepatic YAP/TAZ signaling – in part by interfering with the Notch-YAP feedback loop (Tschaharganeh et al., 2013; Kim et al., 2017b,c). Interestingly, inactivating mutations in the negative Wnt regulator AXIN do not lead to increased Wnt/β-catenin signaling in hepatocellular carcinoma as would be expected, but are instead associated with a proliferative phenotype and gene signatures enriched for Notch and YAP signaling indicating for a Wnt-independent direct or indirect inhibition of YAP by AXIN (Abitbol et al., 2018).

Downstream of activated YAP/TAZ, unchecked proliferation and deregulated cell cycle control are one of the key mechanisms driving tumor development. Several studies showed that YAP/TEAD co-operate with E2F transcription factors downstream of retinoblastoma signaling to promote proliferation in cancer, including in liver cancer (Ehmer et al., 2014; Kapoor et al., 2014; Ehmer and Sage, 2015; Fitamant et al., 2015; Hiemer et al., 2015; Shi et al., 2016). While low levels of active YAP are not sufficient to induce proliferation in quiescent livers, concomitant activation of other pro-proliferative signals in hepatic injury or inflammation give YAP-activated hepatocytes a proliferative advantage leading to their expansion (Ehmer et al., 2014; Su et al., 2015). Aside from proliferation, YAP signaling in HCC is involved in a multitude of cancer-associated pathways, including suppression of apoptosis, deregulated ER/unfolded protein response, and chromosomal instability (CNI) (Rosenbluh et al., 2012; Wu et al., 2015; Weiler et al., 2017). Last, but not least, Hippo signaling is a relevant player in the regulation of a protumoral inflammatory microenvironment. In Mst1/2-deficient livers, the YAP target *Mcp1* triggers accumulation of tumor-infiltrating macrophages that impair immune clearance of transformed hepatocytes and promote HCC development (Guo et al., 2017; Kim et al., 2018). If the expanding role of Hippo signaling in cancer immunity is of any relevance in liver disease remains to be investigated, but could be important in optimizing strategies for HCC-targeted immunotherapies (Taha et al., 2018).

## Targeting Hippo Signaling in Liver Disease

Effective targeting of Hippo signaling *in vivo* – either to promote liver regeneration or to inhibit fibrosis and cancer progression – presents an unmet need in liver disease. Hepatic inactivation of MST kinases or YAP by microencapsulated *siRNA* as well as YAP silencing by AAV-delivered *shRNA* has yielded promising efficiency in murine models, but a transition into clinics is not foreseeable yet (Fitamant et al., 2015; Yin et al., 2016; Loforese et al., 2017; Jiang et al., 2018). Recently, transient activation of YAP/TAZ signaling with improved murine liver regeneration has been achieved by inhibition of MST1/2 kinases using the novel compound XMU-MP-1 (Fan et al., 2016).

In contrast to regeneration, inhibition of YAP/TAZ is a promising therapeutic approach in cirrhosis, steatohepatitis, or hepatocellular carcinoma. Outcomes of YAP activation in the liver are mostly dependent on its interaction with TEAD transcription factors, making YAP/TEAD complexes a promising target. Verteporfin, a substance identified in a compound screen for YAP/TEAD inhibitors, impeded HCC development in *Nf2*-deficient livers (Liu-Chittenden et al., 2012). However, cellular toxicity by off-target effects as well as the production of reactive oxygen radicals upon light activation have hindered the transition of verteporfin into cancer therapy (Konstantinou et al., 2017). Recently, antiparasitic ivermectin was identified as an inhibitor of YAP/TEAD-dependent transcription – and was successfully used to inhibit YAP activation and hepatic overgrowth in Mob1a/1b-deficient livers. While the mechanism of YAP inhibition by ivermectin is not fully understood to date, the compound seems to have a good safety profile, making it a promising drug for *in vivo* YAP inhibition (Nishio et al., 2016). In esophageal adenocarcinoma cells, CA3 – a novel inhibitor of YAP/TEAD-dependent transcription – successfully reduced tumor cell growth together with established chemotherapy (Song et al., 2018). However, this compound has not been tested in the liver so far. In addition to direct YAP/TAZ inhibitors, compounds that target upstream regulators of Hippo signaling have been in the focus of several studies (Bae et al., 2017). In HCC cells, tankyrase inhibitors that upregulate members of the angiomotion family as well as aurokinase inhibitors restricted tumor cell growth by modification of YAP activity (Jia et al., 2017; Liu et al., 2017). While compounds that interfere with mechanotransduction by targeting F-actin or Rho have been successfully used to modify Hippo signaling and YAP activity *in vitro*, their efficiency in the liver cells is not known and the toxicity associated with interference of the cytoskeleton will likely limit their *in vivo* use (Bae et al., 2017). In Wnt-activated colon cancer cells, targeting of YES1 with the approved compound dasatinib inhibits tyrosine phosphorylation of YAP and activation of a β-catenin-YAP1-TBX5 transcriptional complex (Rosenbluh et al., 2012), while in mammary cells inhibition of PI3K/PDK1 was able to restrain the binding of PDK1 to SAV1, resulting disinhibition of upstream Hippo signaling, and reduction of YAP activity (Fan et al., 2013). Again, the efficiency of these compounds in liver cancer has not been investigated and the tissue specificity of YAP-interacting pathways will make the identification of targetable upstream regulators even more complicated. With several promising candidate drugs to inhibit YAP/TEAD being under development, their application is currently limited to preclinical studies and the first clinical trials that are expected in the near future will tell if the immense expectations in targeting YAP activity will hold true in the treatment of human disease.

## Future Perspectives

**FIGURE 2:**
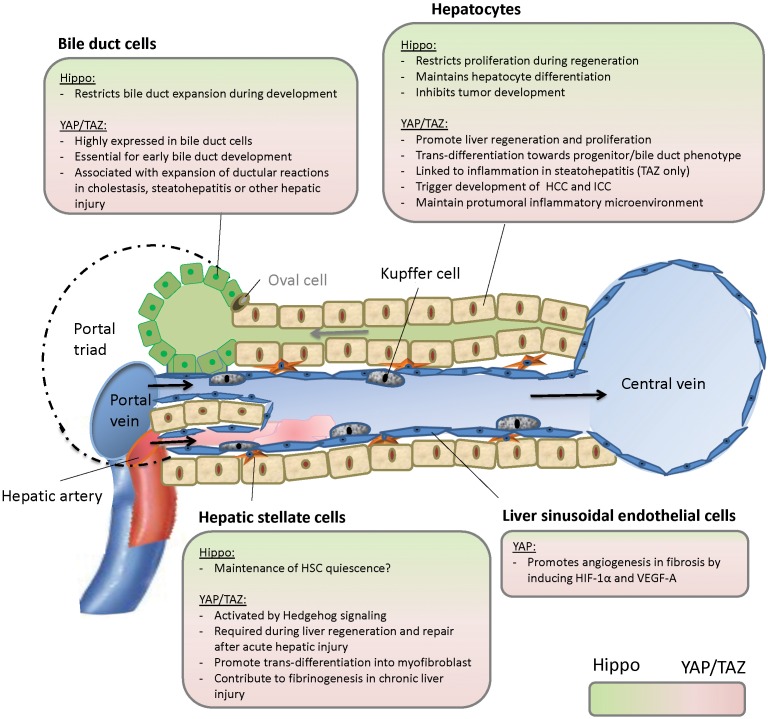
Hippo signaling in the liver. HCC, hepatocellular carcinoma; ICC, intrahepatic cholangiocarcinoma.

Our knowledge about Hippo signaling in different hepatic cell types has expanded over the recent years (summarized in Figure 2), but important questions remain. In chronic and acute liver injury, we still do not fully comprehend the role of Hippo signaling in the interaction between HSC, LSEC, bile duct cells, and hepatocytes. Apart from hedgehog signaling, are there other pathways such as TGFbeta signaling involved and can these pathways be targeted? With therapeutic modification of Hippo signaling still in its infancy, it remains unclear if inhibition of YAP or TAZ in steatohepatitis, cirrhosis, and cancer might be deleterious in underlying liver disease as it could inhibit regeneration upon liver cell damage. It would therefore be more reasonable to target inflammation or tumor specific upstream regulators of Hippo signaling. However, the mechanisms that govern hepatic Hippo pathway regulation during liver development, in liver homeostasis in the adult liver, or in liver disease are not well understood. Additionally, the cell-specific differences in the outcome of YAP and TAZ activation remain to be investigated. It is of special relevance in this context that the role of YAP and TAZ is not completely redundant and deciphering the individual functions of both orthologs will certainly help to understand the role of Hippo signaling in the liver. To approach all these questions in the complex microenvironment of the liver, further studies will be needed.

## Author Contributions

SM and UE drafted and wrote the manuscript.

## Conflict of Interest Statement

The authors declare that the research was conducted in the absence of any commercial or financial relationships that could be construed as a potential conflict of interest.
